# Editorial: Characterization of esophageal cancer molecular signatures and mechanisms using multi-omics analyses

**DOI:** 10.3389/fgene.2023.1334569

**Published:** 2023-11-21

**Authors:** Yuanji Xu, Wei Huang, Amin Tamadon, Yao Lin, Guodong Ye

**Affiliations:** ^1^ Department of Radiation Oncology, Fujian Medical University Cancer Hospital, Fujian Cancer Hospital, Fuzhou, China; ^2^ Fujian Provincial Cancer Hospital, Fuzhou, China; ^3^ Key Laboratory of Radiation Oncology, Shandong Cancer Hospital, Shandong University, Jinan, China; ^4^ PerciaVista R&D Co., Shiraz, Iran; ^5^ Department for Scientific Work, West Kazakhstan Marat Ospanov Medical University, Aktobe, Kazakhstan; ^6^ Fujian University of Traditional Chinese Medicine, Fuzhou, China; ^7^ Xiamen Medical College, Xiamen, China

**Keywords:** esophageal cancer, multi-omics analysis, machine learning, tumor-associated macrophages, Aurora Kinase A

Cancer research is a constantly evolving field, driven by the synergy of cutting-edge technology, meticulous data analysis, and innovative methodologies. In this editorial, we explore recent studies that highlight the potential of modern approaches, such as machine learning and single-cell analysis, to decipher critical factors affecting the prognosis and treatment of various cancers. These groundbreaking insights provide new avenues for the enhancement of cancer diagnosis and the development of more effective therapies.

Esophageal squamous cell carcinoma (ESCC) poses a formidable challenge, demanding innovative insights (Shang et al.). Recent studies have leveraged machine learning techniques, revealing m6A regulators that show promise as prognostic indicators (Shang et al.). YTHDF1 and HNRNPC, in particular, offer a ray of hope for the development of more tailored and efficacious treatments for ESCC (Shang et al.). This urgency for innovative insights is especially palpable in the realm of ESCC, a challenging cancer that requires novel strategies for intervention (Shang et al.).

Tumor-associated macrophages (TAMs) with immunosuppressive properties can impede the effectiveness of immunotherapies (Li et al.). Recent investigations have uncovered a unique subpopulation of TAMs in ESCC, marked by the expression of TREM2 (Li et al.). These TREM2+ TAMs are closely associated with unfavorable clinical outcomes, making them not only potential predictive biomarkers for ESCC prognosis but also catalysts for refining immunotherapy strategies to enhance their effectiveness (Li et al.).

Genetic markers wield a profound influence on cancer susceptibility and prognostic outcomes (Zhong et al.). A recent investigation has delved deep into cell-type-specific expression quantitative trait loci (eQTL) within adenocarcinoma at the gastroesophageal junction (ACGEJ) (Zhong et al.). The results have unearthed a Research Topic of ACGEJ-specific eQTLs, shedding new light on susceptibility and prognosis markers tailored specifically to ACGEJ (Zhong et al.).

AURKA, a pivotal regulator of cell mitosis and tumor progression, remains relatively uncharted territory in terms of its prognostic significance across diverse cancer types (Yang et al.). However, a comprehensive analysis has now revealed that AURKA is prominently overexpressed in the majority of the cancer types under investigation (Yang et al.). This discovery paves the way for further exploration of AURKA’s potential as a predictive biomarker for a wide range of tumors (Yang et al.).

In conclusion, these studies collectively underscore the potential of contemporary analytical techniques to unveil the intricate molecular landscapes of cancer. They provide hope in the ongoing battle for a deeper understanding of cancer and the development of more effective treatments. As we progress in the realm of cancer research, it is crucial to remain vigilant for such groundbreaking discoveries and actively explore their potential clinical applications.

